# Cas-Based Systems for RNA Editing in Gene Therapy of Monogenic Diseases: *In Vitro* and *in Vivo* Application and Translational Potential

**DOI:** 10.3389/fcell.2022.903812

**Published:** 2022-06-16

**Authors:** Vasiliy V. Reshetnikov, Angelina V. Chirinskaite, Julia V. Sopova, Roman A. Ivanov, Elena I. Leonova

**Affiliations:** ^1^ Department of Biotechnology, Sirius University of Science and Technology, Sochi, Russia; ^2^ Department of Molecular Genetics, Institute of Cytology and Genetics, Novosibirsk, Russia; ^3^ Center of Transgenesis and Genome Editing, St. Petersburg State University, St. Petersburg, Russia; ^4^ Laboratory of Amyloid Biology, St. Petersburg State University, St. Petersburg, Russia; ^5^ Scientific Center for Genetics and Life Sciences, Sirius University of Science and Technology, Sochi, Russia

**Keywords:** RNA editing, dCas13, repair, rescue, cure, monogenic disease, gene therapy

## Abstract

Rare genetic diseases reduce quality of life and can significantly shorten the lifespan. There are few effective treatment options for these diseases, and existing therapeutic strategies often represent only supportive or palliative care. Therefore, designing genetic-engineering technologies for the treatment of genetic diseases is urgently needed. Rapid advances in genetic editing technologies based on programmable nucleases and in the engineering of gene delivery systems have made it possible to conduct several dozen successful clinical trials; however, the risk of numerous side effects caused by off-target double-strand breaks limits the use of these technologies in the clinic. Development of adenine-to-inosine (A-to-I) and cytosine-to-uracil (C-to-U) RNA-editing systems based on dCas13 enables editing at the transcriptional level without double-strand breaks in DNA. In this review, we discuss recent progress in the application of these technologies in *in vitro* and *in vivo* experiments. The main strategies for improving RNA-editing tools by increasing their efficiency and specificity are described as well. These data allow us to outline the prospects of base-editing systems for clinical application.

## Gene Therapy Approaches

More than 100 million persons are affected by rare genetic diseases globally (Nguengang Wakap et al., 2020). These diseases have high mortality and morbidity rates and often require specialized healthcare services and treatments; therefore, the management of these diseases is a large socioeconomic burden (Ferreira, 2019). As a possible model for the treatment of genetic diseases caused by the lack of functional activity or by complete absence of a protein, various technologies are used based on the delivery of a genetic construct having the correct gene sequence (gene addition strategies) *via* chemical, physical, or viral delivery (Giacca and Zacchigna, 2012; Kotterman et al., 2015; Prakash et al., 2016; Salameh et al., 2020; Konishi and Long, 2021). Delivery of genetic tools by means of viral vectors is widely used in clinical trials and is considered the most promising administration route featuring low cytotoxicity, good transfection efficiency, and high efficacy (Ginn et al., 2018). Among the many viral vectors [lentiviral, adenoviral, retroviral, and adeno-associated-virus (AAV)-based], it is AAV viruses (characterized by low immunogenicity and cytotoxicity) that are most often employed in clinical trials and were recently approved for the treatment of inherited blindness and spinal muscular atrophy (Ginn et al., 2018; Wang et al., 2019a; Li and Samulski, 2020).

Aside from gene addition strategies, there have been developments in the technology of gene editing by means of programmable nucleases: zinc-finger nucleases, transcription activator–like effector nucleases (TALENs), and CRISPR-associated (Cas) proteins. All these tools are utilized in clinical trials (including phase II–III trials) for the treatment of various genetic diseases (Cui et al., 2021; Guo et al., 2021). The most rapidly developing gene-editing system is CRISPR–Cas [for comprehensive review see (Leonova and Gainetdinov, 2020; Guo et al., 2021)], which was first used for mammalian genome editing in 2013 (Cong et al., 2013). In addition to genome editing, Cas-based tools can also help to regulate gene expression. Fusion of catalytically inactive RNA-targeting enzyme dCas13b—either with one of adenosine deaminases from the ADAR family or with cytosine deaminase APOBEC3A—forms the basis for RNA-editing tools (Cox et al., 2017; Matharu et al., 2019).

G-to-A and C-to-T mutations are common in mammals and represent ∼61% of all point mutations annotated in the ClinVar database (Rees and Liu, 2018). RNA editing also allows to implement protein recording and alters alternative splicing sites because inosine is recognized by the tRNA system as guanidine. Additionally, RNA editing can be utilized for improving microRNA specificity or RNA stability or for helping RNAs to assume their secondary structures (Kung et al., 2018). Interestingly, in bacteria there is only one example of A-to-I editing in mRNA of *hokB* toxin gene that can regulate growth arrest and antibiotic sensitivity (Bar-Yaacov et al., 2018). In the present review, we will look at examples of applications of genetic-engineering systems for site-directed RNA editing for therapy of monogenic diseases *in vitro* and *in vivo*.

## Delivery Methods and Targeting Considerations for RNA Editing Systems

Gene therapy is the only effective modality for the treatment of many rare hereditary diseases. According to the PubMed database, the number of gene therapy studies has more than tripled in the last 20 years. Despite active research in the field of gene therapy, there are still many unsolved problems that limit the use of such tools in clinical practice. A common problem with gene addition and gene-editing strategies is finding efficient and safe ways to deliver the genetic constructs to a target organ. Even though already three gene therapies based on AAV vectors (Glybera, Luxturna, and Zolgensma) are approved by the FDA (Scott, 2015; Al ‐ Zaidy et al., 2019; Maguire et al., 2019), and AAVs themselves have low immunogenicity, cytotoxicity, and high tropism for target tissues, there are still cases of serious complications (even death) caused by AAV-based treatments (Wilson and Flotte, 2020; Arnold, 2021). Short-term complications may be related to adverse effects of high doses of AAVs (Wilson and Flotte, 2020). On the other hand, long-term complications may be induced by unintended integration of a viral vector into the genome, resulting in a change in the expression of host genes and the risk of malignant cell transformation (Donsante et al., 2001; Donsante et al., 2007; Nguyen et al., 2021). Different AAV serotypes have diverse tropism to various human tissues due to a variety of cellular receptors (Lisowski et al., 2015). The delivery of large gene sequences *via* viral vectors is complicated, because for AAV limitation of packaging capacity is under ∼5 kbp DNA and for high-capacity adenoviral vectors - under ∼36 kbp DNA (Wang et al., 2019a; Li and Samulski, 2020). Accordingly, major research efforts are now devoted to capsid engineering in AAV vectors with the aim of enhancing tropism to target tissues (including rational design (Müller et al., 2003), directed evolution (Ojala et al., 2018) and chemical conjugation (Liu et al., 2013), reducing immunogenicity (humoral and cellular immunity against AAV), and improving the *trans*-splicing technology to increase packaging capacity (Srivastava, 2016; He et al., 2021). In addition, nonviral delivery technologies based on lipids, lipid-like nanomaterials, or gold nanoparticles are being designed too (Lee et al., 2017; Rui et al., 2019; He et al., 2021). Delivery of genetically engineered constructs *via* lipid nanoparticles has been approved for a clinical trial (NCT04601051) (Gillmore et al., 2021). Moreover, a few years ago, the first small-interfering-RNA therapeutic packaged into nanoparticles (patisiran) was approved by the FDA for the treatment of hereditary amyloidogenic transthyretin amyloidosis (Kulkarni et al., 2019). Disadvantages of the nonviral methods include low specificity to target tissues and short half-life in the systemic circulation. Altogether, the choice of a technique for delivery to cells and the route of administration (intravenous, intraperitoneal, or targeted administration to an organ) mostly determine the severity of adverse effects and the treatment outcome. Therefore, despite the adequate characteristics of genetically engineered constructs, introduction of gene therapies into clinical practice requires both long-term preclinical *in vivo* experiments on animals and long-term monitoring of participants of clinical trials.

## Evolution of Cas13-Based RNA-Editing Systems

**FIGURE 1:**
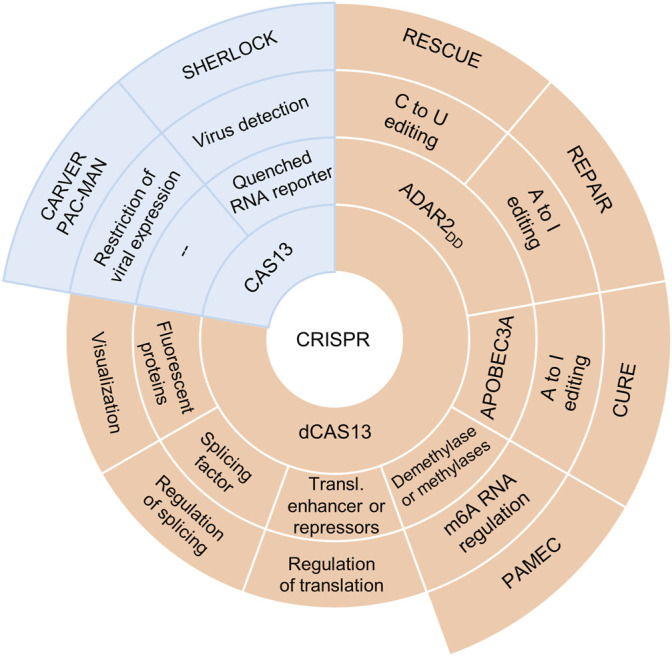
A brief overview of Cas13-based applications. For the detection of viral genomes *in vitro*, CRISPR–Cas13a is used as part of specific high-sensitivity enzymatic reporter UnLOCKing (SHERLOCK), where CRISPR–Cas13a cleaves a target RNA in the presence of a quenched RNA reporter that emits its fluorescence signal (Gootenberg et al., 2018). CRISPR–Cas13 is also a component of the CARVER system (Cas13-assisted restriction of viral expression and readout), which is aimed at specific degradation of viral RNA *in vivo* and has been successfully applied to various viral RNAs in cultured cells (Freije et al., 2019). Moreover, CRISPR–Cas13d within the PAC-MAN (prophylactic antiviral CRISPR in human cells) system inhibits SARS-CoV-2 replication by directly targeting and cleaving all viral positive-sense RNA (Abbott et al., 2020). The fusion of CRISPR–dCas13 with florescent proteins helped to use this system for RNA imaging and research on trafficking of RNAs (Abudayyeh et al., 2016); fusion with a splicing factor allows for regulation of alternative pre-mRNA splicing (Wang et al., 2019b), whereas fusion with a translational enhancer or repressor can help to manage the translation of a specific mRNA (Abudayyeh et al., 2016). Some investigators (Zhao et al., 2020) devised a photoactivatable RNA-m^6^A-editing system using CRISPR–dCas13 (PAMEC), which enables regulation of the methylation level of target RNA sites by means of light of different wavelengths. The fusion of dCas13 with deaminases has laid the foundation for the creation of site-directed RNA base–editing systems CURE, REPAIR, and RESCUE.

The complex of the Cas13 nuclease with RNA containing clustered regularly interspaced short palindromic repeats (crRNA) is the foundation of the CRISPR–Cas13 system, which can specifically bind to single-strand RNA and cleave it. In bacteria and archaea, the CRISPR–Cas13 system is a component of the adaptive immune system, which performs programmable RNA-guided degradation of foreign RNAs. Four phylogenetically distinct variants of Cas13 have been identified so far: Cas13a (previously known as C2c2), Cas13b, Cas13c, and Cas13d. All these nucleases have two nucleotide-binding domains (HEPNs), which are required for pre-crRNA processing and for the cleavage of a target single-stranded RNA (Abudayyeh et al., 2017; O'Connell, 2019). The CRISPR–Cas13 system has found many applications in molecular biology (Ashraf et al., 2022). One exotic application is the delivery of carrier phage capsid with packaged CRISPR-Cas13a targeted against antibiotic resistance gene, into bacterial cells (Kiga et al., 2020). Not so long ago, progress in genetic engineering helped to create a Cas13b variant devoid of RNase activity—dCas13b—which is still capable of RNA binding (Cox et al., 2017). This was made possible by replacement of two histidines with alanines (mutations H133A and H1058A) in HEPN domains. RNA-binding programmability by means of CRISPR–dCas13 has significantly expanded its applicability (Cox et al., 2017; O'Connell, 2019; Ashraf et al., 2022). CRISPR–dCas13-based systems have also found diverse basic-research and clinical applications (Figure 1).

One of the most promising areas of practical application of CRISPR–dCas13 from the standpoint of medical treatments is the development of the site-directed RNA editing (SDRE) system. This accomplishment has resulted from the fusion of CRISPR–dCas13 with an adenosine or cytosine deaminase (ADAR or APOBEC3A), which is capable of replacing adenosine with inosine (A to I) or cytosine with uracil (C to U) (Cox et al., 2017; Matharu et al., 2019). In this way, CRISPR–dCas13 allows to focus the catalytic activity of ADAR or APOBEC3A on a certain adenosine or cytosine (in an mRNA sequence) that has undesirable consequences (alterations in a splicing site, loss of functional activity of a microRNA, amino acid substitution, or premature termination of translation).

**FIGURE 2:**
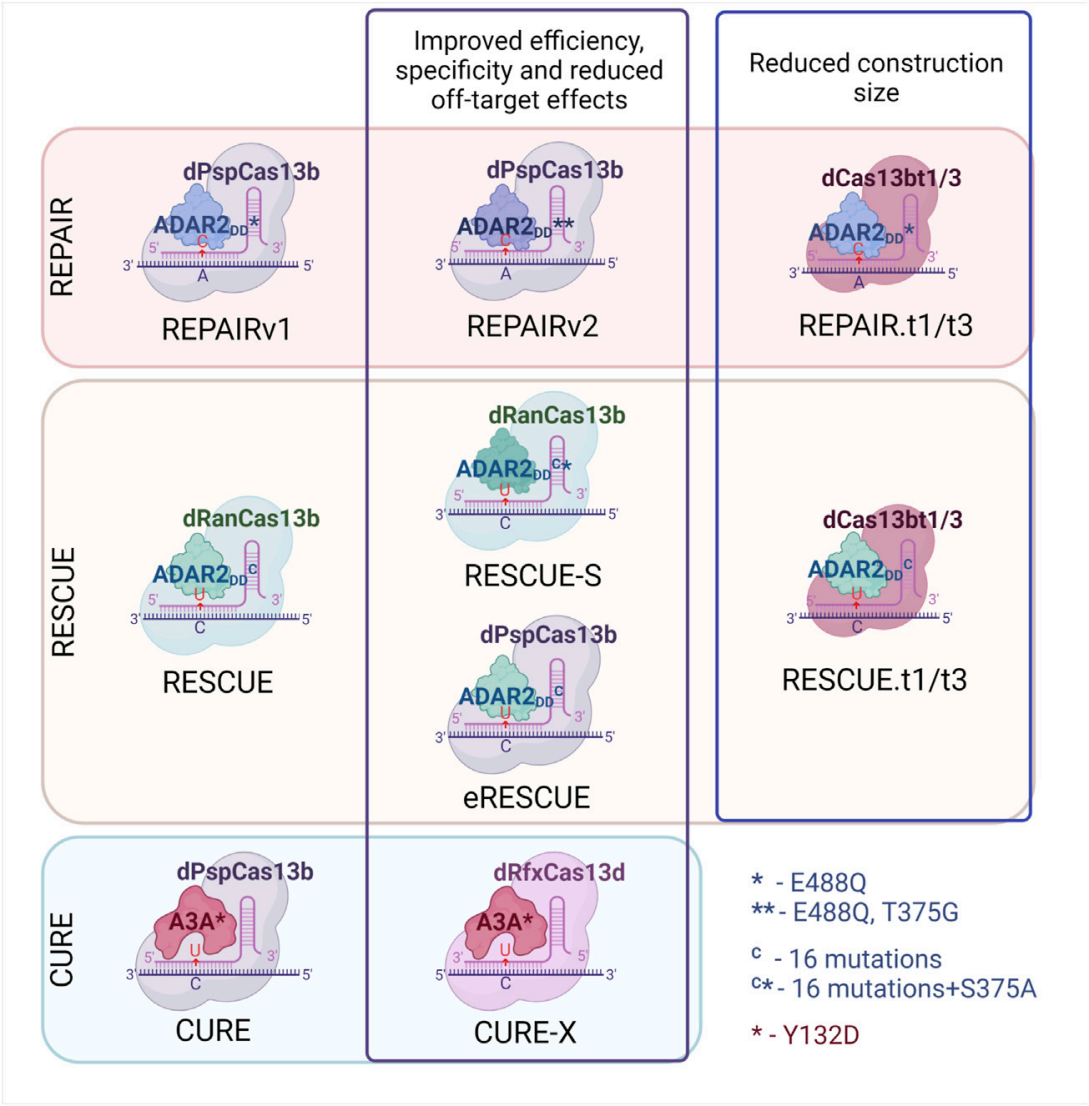
Development of site-directed Cas13-based RNA-editing systems. Development of REPAIR (A-to-I) RNA editing system, RESCUE and CURE (C-to-U) editing systems. Explanation see in the text.

The first CRISPR–Cas-targeted RNA-editing system was developed in 2017 (Cox et al., 2017). By fusing dCas13b from *Prevotella* sp. (hereafter referred to as dCas13b) with the deaminase domain of ADAR2 (E488Q) (hereafter referred to as ADAR2_DD_), they created RNA Editing for Programmable A to I Replacement version 1 (REPAIRv1). This system was successfully utilized for the editing of mutations in cell culture, and editing efficiency reached 35%. A major drawback of this system is a large number of off-target effects. To solve this problem, a modified ADAR2 system with two amino acid substitutions has been created: ADAR2_DD_ (E488Q/T375G). Fusion of dCas13b with ADAR2DD (E488Q/T375G) has been incorporated into REPAIRv2, the use of which in cell culture *in vitro* has reduced the number of off-target events from 18,385 (REPAIRv1) to 20 (REPAIRv2) according to transcriptomic analysis, confirming significantly higher specificity of the new system (Figure 2). At the same time, conversion efficiency remains approximately at the same level (Cox et al., 2017).

Introducing 16 mutations into ADAR2_DD_ allowed to expand the functional activity of this deaminase: aside from adenosine deamination, the enzyme now can carry out cytosine deamination with C-to-U conversion and G-to-A functional substitution (Abudayyeh et al., 2019). This mutated ADAR2_DD_ was fused with catalytically inactive *Riemerella anatipestifer* Cas13 (hereafter referred to as dRanCas13b), and the resulting system was named RNA Editing for Specific C to U Exchange (RESCUE) (Abudayyeh et al., 2019). Nonetheless, the risk of unintended transcriptomic modifications also got higher due to the extra deamination activity. To solve this problem, ADAR2_DD_ was mutated again, and the introduction of the S375A mutation helped to diminish the number of C-to-U off-target effects approximately 1.8-fold and A-to-I off-target effects almost 12-fold while maintaining specificity. This system was named RESCUE-S. Despite the appealing applications of such a versatile RNA-editing system, the effectiveness of RNA editing in a β-catenin transcript (*CTNNB1*) turned out to be lower than that of other systems and did not exceed 15%.

To improve the RESCUE system, investigators used a Cas13b ortholog: dPspCas13b (instead of dRanCas13b) with a nuclear export sequence (NES) (Li et al., 2021). This system is called eRESCUE. In multiple cellular transcripts, both C-to-U editing and A-to-I editing with eRESCUE are up to 2-fold more efficient as compared to the dRanCas13b-RESCUE-NES system. Nevertheless, off-target activity of the eRESCUE system is higher than that of dRanCas13b-RESCUE-NES (Li et al., 2021).

Apart from mutated ADAR2_DD_ with cytidine deaminase activity, in mammals there is a cytidine deaminase APOBEC3A (A3A), which can act on both RNA and DNA substrates (Sharma et al., 2015). Huang and others fused this deaminase with dPspCas13b and developed a C-to-U editing tool named CURE (C to U RNA Editor) (Huang et al., 2020). A3A has high specificity for UC dinucleotides; hence, the off-target activity of this system is quite low. Guide RNAs (gRNAs) are designed to create loops at target sites because A3A is active in specific loop regions. Off-target editing was further reduced by the attachment of a NES to dCas13. Another version of the CURE system—CURE-X—involves another nuclease, CasRx (also known as RfxCas13d), instead of Cas13 to reduce off-target editing. These systems show great variation in RNA editing efficiency and in off-target activity, and in some cases, effectiveness is higher than that of RESCUE-S (Huang et al., 2020).

To take advantage of these protein systems in medical treatments, the sequences should be delivered into cells using, for example, AAV vectors. Due to the long amino acid sequence of Cas13, some researchers (Cox et al., 2017) had to cut ADAR2_DD_ to fit it into AAV. Another way to overcome this problem is to find smaller Cas13 proteins. Some authors (Kannan et al., 2021) analyzed more than 5000 bacterial genomes and discovered novel subfamilies of small Cas13 proteins (∼800 aa in comparison with ∼1100 aa Cas13a) within Cas13b and Cas13c subtypes (Cas13bts and Cas13cts, respectively). Three proteins, Cas13bt1, Cas13bt2, and Cas13bt3 were characterized, and two of them (Cas13bt1 and Cas13bt3) were fused with ADAR2_DD_; the resultant systems are called REPAIR.t1 and REPAIR.t3, respectively. Similarly to REPAIR.t1 and REPAIR.t3, the fusion of Cas13bt1 or Cas13bt3 with modified ADAR2_DD_ gave rise to RESCUE.t1 and RESCUE.t3, respectively. The efficiency of REPAIR.t1/t3 and RESCUE.t1/t3 is comparable to that of the previously described systems. Overall, improvements in site-directed RNA-editing systems are currently focused on increasing the efficiency of deaminases, on lowering the number of off-target events, and on reducing the size of the genetic construct to facilitate *in vivo* delivery.

## Site-Directed RNA Base Editing for Monogenic-Disease Therapy

Even though dCas13-based site-directed RNA-editing systems were discovered only recently, successful results have already been obtained in the treatment of monogenic diseases in cell culture models. The first successful practical application of RNA editing was implemented using the REPAIR system (Cox et al., 2017). Into HEK293FT cells, researchers transfected expression constructs for cDNA of genes carrying the 878G > A (*AVPR2* W293X) mutation, which is associated with X-linked nephrogenic diabetes, and the 1517G > A mutation (*FANCC* W506X), which is associated with Fanconi anemia (Table 1). Missense mutation W293X leads to a nonfunctional type 2 receptor and insensitivity of cells to changes in arginine vasopressin concentration in the blood (Wildin et al., 1998). Missense mutation W506X impairs DNA repair because of reduced activity of the FANCC protein, thereby causing cytogenetic instability, hypersensitivity to DNA-crosslinking agents, and increased chromosomal breakage (Liu et al., 1999). REPAIRv1 successfully corrected the mutations, with an editing efficiency of 35% for the *AVPR2* gene and 23% for *FANCC* (Cox et al., 2017). Of note, the entire REPAIRv1 genetic construct was packaged into AAV vectors, which were used for delivery. Given that AAV vectors are currently regarded as most applicable to therapeutic uses, REPAIRv1 is expected to further minimize the problems of construct delivery to a target organ.

**TABLE 1:** RNA editing for therapy of monogenic diseases in mouse models.

Strain	Model	Delivery system	gRNA delivery system	Editing system	Target	Tissue	References
*In vivo*							
Mecp^2317G>A ^mice	Rett syndrome	AAV	λN-BoxB	hADAR2 (E488Q)	*Mecp2*	Hippocampus	Sinnamon et al. (2020)
Mdx mice	Duchenne muscular dystrophy	AAV	MS2 GluR2 transcript	hADAR2 (E488Q)	*Mdx*	Muscle	Katrekar et al. (2019)
sp^fash^ mice	Ornithine transcarbamylase deficiency	AAV	GluR2 transcript	hADAR2 (E488Q)	*Otx*	Liver	Katrekar et al. (2019)
Idua-W392X mice	Hurler syndrome	AAV	cadRNAs	hADAR2 (E488Q)	*IDUA*	Liver	Katrekar DY et al. (2021)
*In vitro*/*Ex vivo*							
Neuro2A cells, Primary neurons from Mecp2^R106Q^ mice	Rett syndrome	AAV	λN-BoxB	hADAR2 (E488Q)	*Mecp2*	--	Sinnamon et al. (2017)
HEK293T cells	Monogenetic type of Parkinson’s disease	Plasmid	--	ADAR gRNAs	*PINK1*	--	Wettengel et al. (2017)
Primary fibroblasts from Hurler syndrome patient	Hurler syndrome	Plasmid	--	ADAR gRNAs	*IDUA*	--	Qu et al. (2019)
HEK293FT cells	X-linked nephrogenic diabetes	AAV	dCas13	hADAR2 (E488Q)	*AVPR2*	--	Cox et al. (2017)
HEK293FT cells	Fanconi anemia	AAV	dCas13	hADAR2 (E488Q)	*FANCC*	--	Cox et al. (2017)
HEK293T cells	Usher syndrome type 2	Plasmid	dCas13	hADAR2 (E488Q)	*USH2A*	--	Fry et al. (2020)

One research group (Fry et al., 2020) used the REPAIRv2 system to edit the c.11864G > A nonsense mutation in the Usher Syndrome 2A (*USH2A*) gene. USH2A is important in the development and homeostasis of the inner ear and retina, whereas the absence of the functional protein leads to sensorineural hearing loss and retinitis pigmentosa. Sequence of exon 60 of *USH2A* cDNA carrying the c.11864G > A mutation was delivered *via* a plasmid into HEK293T cells along with the REPAIRv2 construct. The efficiency of target adenosine editing was 43%.

Thus, successful application of site-directed Cas13-based RNA-editing systems has been demonstrated for RNA editing and for restoration of a protein product’s function (Cox et al., 2017; Fry et al., 2020). Preliminary data (Rashnonejad et al., 2019) also indicate that CRISPR–Cas13 can successfully suppress *DUX4* mRNA translation. Normally, DUX4 is expressed only in the embryonic period, whereas inadequate DUX4 repression in the postnatal period is associated with progressive muscle wasting and weakness (facioscapulohumeral muscular dystrophy). By means of several Cas13b–gRNAs targeted to different parts of *DUX4* mRNA, those authors achieved >90% downregulation of the DUX4 protein in experiments on cultured cells and a near 100% reduction of DUX4 expression in tibialis anterior muscles of the TIC-DUX4 mouse model.

In addition to dCas13-ADAR2_DD_ RNA-editing systems, other RNA-editing systems are currently being actively developed, such as BoxB-λN-ADAR_DD_, SNAP-tag-ADAR_DD_, and MCP-MS2-ADAR_DD_, in which the binding of ADAR_DD_ and gRNA is implemented by the λN peptide, SNAP-tag protein, and MS2 bacteriophage coat-binding protein, respectively (Fry et al., 2020; Khosravi and Jantsch, 2021). BoxB-λN-ADAR_DD_ has manifested greater efficiency of editing of point mutations in *Mecp* mRNA *in vivo* (∼50%) and *in vitro* (∼70%) (Sinnamon et al., 2017; Sinnamon et al., 2020). Mutations in the *Mecp* gene are the etiology of Rett syndrome. Treatment of Duchenne muscular dystrophy in the Mdx mouse model *via* editing of the mutation in the *Mdx* gene by means of the MCP-MS2-ADAR_DD_ system yielded less impressive results (up to 3.6% of edited RNA and up to 2.5% protein restoration) (Katrekar et al., 2019). Similarly, dCas13-based SDRE BoxB-λN-ADAR_DD_ and MCP-MS2-ADAR_DD_ systems are relatively small and can be packaged into AAV vectors.

The second important family of editing tools is based on ADAR gRNA, whose structure mimics ADAR substrates, thereby ensuring the recruitment of exogenous and/or endogenous ADARs; the presence of a programmable antisense region that is complementary to the target RNA sequence affords “site-directed” RNA editing (Fry et al., 2020; Kannan et al., 2021). Among approaches involving endogenous ADARs, it is worth mentioning the RESTORE system (Recruiting Endogenous ADAR to Specific Transcripts for Oligonucleotide-mediated RNA Editing), which is based on short chemically modified antisense oligonucleotides, and LEAPER (Leveraging Endogenous ADAR for Programmable Editing of RNA), which involves long antisense RNAs (Merkle et al., 2019; Qu et al., 2019). ADAR gRNAs often use the naturally occurring R/G motif of GluR2 sequence or artificially synthesized sequences. These approaches are responsible for the substantial progress in the editing of mutant *IDUA* pre-mRNA both *in vitro* (efficiency up to 80%) (Qu et al., 2019) and *in vivo* (efficiency up to 17%) in Idua-W392X mice (Katrekar DY et al., 2021). A nonsense mutation in the *IDUA* gene leads to the absence of an enzyme (α-l-iduronidase) and the buildup of large sugar molecules (glycosaminoglycans) in lysosomes, resulting in one form of type 1 mucopolysaccharidosis (Hurler syndrome). Despite relatively low magnitude of RNA editing in the liver of Idua-W392X mice (Katrekar DY et al., 2021) (≤17%), this was sufficient for a 50% reduction of the glycosaminoglycan accumulation in lysosomes. Other studies on ADAR gRNA have shown restoration of ornithine transcarbamylase function (up to 34% of edited RNA and ≤2.5–5.0% protein restoration) in the liver of ornithine transcarbamylase–deficient spf^ash^ mice (Katrekar et al., 2019) and successful editing of the mutant RNA of the *PINK* gene (up to 65%) in cultured HEK293T cells; the loss of the functional protein product of this gene is associated with the development of the monogenetic type of Parkinson’s disease (Wettengel et al., 2017).

One of the reasons for the scarcity of studies on Cas13-based RNA-editing systems aimed at correcting the effects of genetic mutations may be that these systems were discovered relatively recently. Other approaches to RNA editing have yielded considerable progress in in vivo experiments and therefore offer a wide variety of experimental models for evaluating the effectiveness of CRISPR–Cas13-based systems. The most important characteristics for clinical dissemination of SDRE systems are their effectiveness and specificity (a low number of off-target events). Although it is not yet possible to compare different RNA-editing systems by these parameters, it should be mentioned that the studies in question (Cox et al., 2017; Fry et al., 2020) were performed on cultured cell lines with forced expression of fragments of mutant genes. The performance of such systems *in vivo* can be much different. Furthermore, the percentage of edited RNA is often higher than the percentage of the protein with a restored function. Consequently, new studies are needed to evaluate the effectiveness of CRISPR–Cas13-based RNA-editing systems *in vivo*.

## Translational Potential of Site-Directed RNA-Editing Systems for Gene Therapy of Monogenic Diseases

The most important safety limitation is the off-target effects, which inevitably arise when an SDRE is employed (Mao et al., 2019). Strategies for lowing the number of off-target events in dCas13-based SDRE include (i) introduction of point mutations into the deaminase domain for increasing deamination specificity, (ii) selection of programmable binding proteins that are not promiscuous, and (iii) unconventional configuration of the fusion protein, which may impair deaminase–RNA interaction *via* steric hindrance, thereby reducing the off-target effects (Mao et al., 2019). Substantial progress in the engineering of genetic editors has already allowed to create stable system (REPAIRv2) with a minimal number of off-target events. A reduction in this number is usually proportional to a reduction in editing efficiency; accordingly, one of the urgent tasks is to optimize existing editors in order to achieve the best ratio of efficiency to off-targets. It should be pointed out that the number of off-target events and the efficiency of editing also depend on the uniqueness of their target RNA sequence. Lately, relevant tools have been coming out (created *via* deep-learning–based computational modeling) that evaluate the effectiveness of editing (Song et al., 2020).

The applicability scope of basic RNA editors is limited by the finding that they can correct the effects of only ∼60% of pathogenic point mutations. CRISPR–Cas13-based tools along with a microRNA or antisense oligonucleotide can be successfully employed to treat dominant mutations (e.g., polyglutamine diseases, including Huntington’s disease, spinobulbar muscular atrophy, dentatorubral-pallidoluysian atrophy, and several spinocerebellar ataxias) (Matos et al., 2018; Silva et al., 2020). SDRE systems do not affect DNA sequence, and consequently their effects can be considered temporary and reversible; thus, clinical use of these tools may be more attractive than that of gene-editing tools. The duration of action of RNA editors in the cell is mainly influenced by the delivery method: a ribonucleoprotein complex rapidly degrades in the cell, whereas AAV vector genomes in episomal form can persist in the cell for several years (Niemeyer et al., 2009; Nathwani et al., 2011a; Nathwani et al., 2011b; Lin et al., 2022). Notably, the rapid degradation of Cas-based editors in the form of a ribonucleoprotein complex significantly diminishes off-target effects (Doman et al., 2020). Thus, for diseases that require short-term RNA correction (for example, during a critical period of child development), SDRE would be optimal. AAV-mediated delivery of an SDRE system may be utilized for long-term therapy of an inherited disease. An important prerequisite is stable expression of the vector genomes, which reduces the risk of the need for repeated treatment. Apart from financial implications, the repeated administration of AAV vectors may entail transduction suppression by an increased titer of a neutralizing antibody (Guggino et al., 2020). Consequently, RNA editors can abrogate the effects of a wide range of mutations, and these editors have advantages and disadvantages as compared to CRISPR–Cas9 or base editing; the choice of treatment in each case depends on characteristics of the genetic disease in question.

## Conclusion

Incessant improvement of CRISPR–Cas-based RNA-editing systems and of delivery modalities is step by step bringing about the era when rare mutations will not mean a death sentence for their carriers. On the other hand, at present, there is a large gap between basic research and translation into therapies for rare diseases (Tambuyzer et al., 2020). One of the main goals of the International Rare Diseases Research Consortium is the acceptance of 1000 new therapies for rare diseases into clinical practice by 2027 (Austin et al., 2018), among which gene therapy is expected to occupy an important place. Still, as recent events suggest, it is dangerous to accelerate the design of gene therapies; hence, their safety should be a top priority. Exceptions may only be made if a gene therapy can be regarded as a treatment of last resort (salvage therapy). In terms of safety, Cas-based RNA editors are promising, because as already noted, these systems do not induce double-strand DNA breaks. Nevertheless, to use these editors in clinical practice, it is necessary to conduct systematic preclinical studies on rodents, nonhuman primates, and other animal models that would allow for the evaluation of delayed adverse effects. Besides, systematic investigation is needed to select optimal combinations of conditions (e.g., the editor type, delivery conditions, vector concentration, and neoadjuvant therapy). Altogether, these comprehensive studies will make an invaluable contribution to the progress of gene therapy.
